# *N*-[4-(7-Meth­oxy-2-oxo-2*H*-chromen-8-yl)-2-methyl­butan-2-yl]propionamide

**DOI:** 10.1107/S1600536811033149

**Published:** 2011-08-27

**Authors:** L. Amirthasanjeevi, K. Ravi Kumar, S. S. Rajan

**Affiliations:** a Centre of Advanced Study in Crystallography and Biophysics, University of Madras, Guindy Campus, Chennai 600 025, India; b Laboratory of X-ray Crystallography, Indian Institute of Chemical Technology, Hyderabad, Andhra Pradesh 500 607, India; c Department of Biosciences, Sri Sathya Sai University, Vidya Giri, Puttaparthi, Andhra Pradesh 515 134, India

## Abstract

In the crystal structure of the title osthol derivative, C_18_H_23_NO_4_, mol­ecules are linked by N—H⋯O hydrogen bonds into an infinite chain running parallel to the *c* axis. The CH_3_CH_2_– atoms of the propionamide group are disordered over two sets of sites with refined occupancies of 0.689 (12) and 0.311 (12).

## Related literature

For the synthesis of the title compound, see: Ritter & Minieri (1948 ▶). For the crystal structure of the parent compound osthol [systematic name: 7-meth­oxy-8-(3-methyl­but-2-en­yl)-2-chromenone], see: Borowiak & Wolska (1989 ▶). For biological applications of osthol and its derivatives, see: Liu *et al.* (1998 ▶, 2005 ▶); Okamoto *et al.* (2007 ▶); Huang *et al.* (1996 ▶). For standard bond lengths, see: Allen *et al.* (1987 ▶).
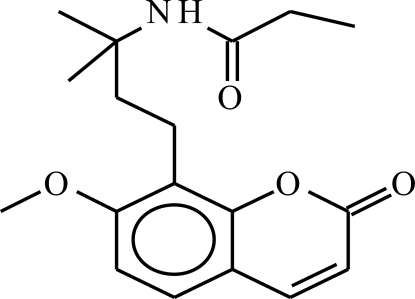


## Experimental

### 

#### Crystal data

C_18_H_23_NO_4_
*M* *_r_* = 317.37
Monoclinic, 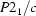

*a* = 11.3555 (11) Å
*b* = 15.5452 (15) Å
*c* = 9.7642 (10) Å
β = 95.617 (2)°
*V* = 1715.3 (3) Å^3^
*Z* = 4
Mo *K*α radiation
μ = 0.09 mm^−1^
*T* = 273 K
0.22 × 0.20 × 0.20 mm

#### Data collection

Bruker SMART APEX CCD area-detector diffractometer
Absorption correction: multi-scan (*SADABS*; Sheldrick, 1996 ▶) *T*_min_ = 0.981, *T*_max_ = 0.983
16205 measured reflections
3019 independent reflections
2537 reflections with *I* > 2σ(*I*)
*R*_int_ = 0.019

#### Refinement

*R*[*F* ^2^ > 2σ(*F* ^2^)] = 0.045
*wR*(*F* ^2^) = 0.136
*S* = 1.01
3019 reflections
234 parameters
H atoms treated by a mixture of independent and constrained refinement
Δρ_max_ = 0.16 e Å^−3^
Δρ_min_ = −0.13 e Å^−3^

### 

Data collection: *SMART* (Bruker, 2001 ▶); cell refinement: *SAINT-Plus* (Bruker, 1999 ▶); data reduction: *SAINT-Plus*; program(s) used to solve structure: *SHELXS97* (Sheldrick, 2008 ▶); program(s) used to refine structure: *SHELXL97* (Sheldrick, 2008 ▶); molecular graphics: *ORTEPIII* (Burnett & Johnson, 1996 ▶); software used to prepare material for publication: *SHELXL97* and *PARST* (Nardelli, 1995 ▶).

## Supplementary Material

Crystal structure: contains datablock(s) I, global. DOI: 10.1107/S1600536811033149/kj2174sup1.cif

Structure factors: contains datablock(s) I. DOI: 10.1107/S1600536811033149/kj2174Isup2.hkl

Supplementary material file. DOI: 10.1107/S1600536811033149/kj2174Isup3.cml

Additional supplementary materials:  crystallographic information; 3D view; checkCIF report

## Figures and Tables

**Table 1:** Hydrogen-bond geometry (Å, °)

*D*—H⋯*A*	*D*—H	H⋯*A*	*D*⋯*A*	*D*—H⋯*A*
N1—H1*N*⋯O4^i^	0.87 (2)	2.10 (2)	2.9546 (15)	169.1 (15)

Symmetry code: (i) 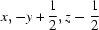.

**Fig. 1.**
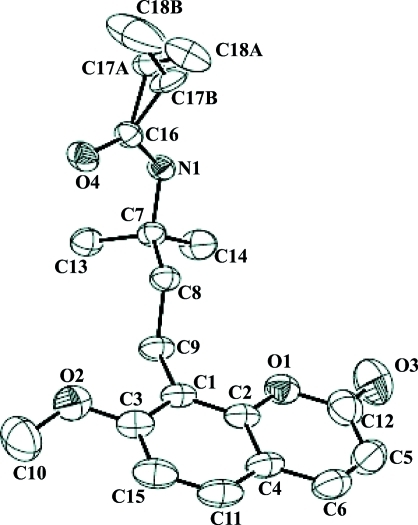
ORTEP plot of the molecule with 30% probability displacement ellipsoids and atom numbering scheme.

**Fig. 2.**
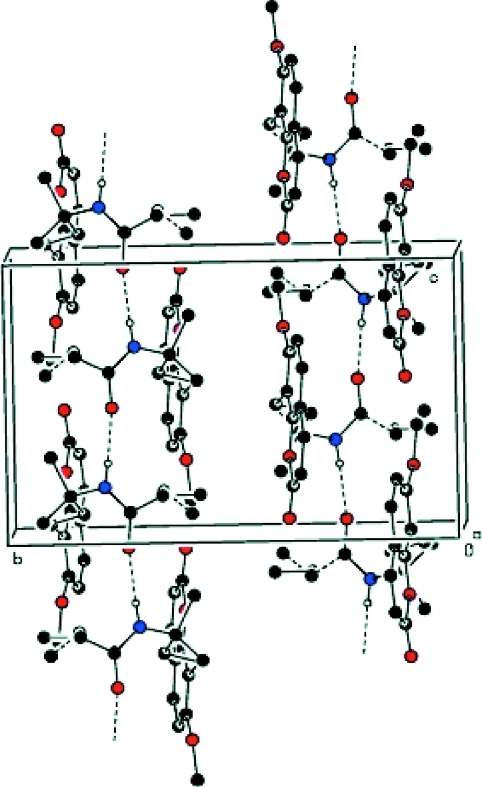
Crystal packing viewed down the a axis.

## supplementary crystallographic information

### Comment

Osthol, isolated from Imperatoria Osthruthium, exhibits anti-inflammatory activity in rats (Liu *et al.*, 2005) and is used in a variety of traditional medicinal preparations in India and China (Liu *et al.*, 1998). A few synthetic derivatives of osthol are used in hepatitis prevention (Okamoto *et al.*, 2007) since osthol specifically increases the glycosylation of the hepatitis antigen and secretion of hepatitis-B virus *in vitro* (Huang *et al.*, 1996). In this paper we report the synthesis and crystal structure of a semi-synthetic derivative of osthol. The compound was prepared by a Ritter reaction (Ritter & Minieri, 1948) and the resulting derivative contains a single bond instead of a double bond at the isoprenyl unit of osthol. The final product also contains a propionamide group (Fig. 1).

The bond lengths and bond angles of the present Osthol derivative are close to the reported values except for the bond angles at C7. The  observed bond angles at C7 (C8—C7—C13 = 110.6 (1)° and C8—C7—C14 = 112.2 (1)°) deviate significantly from the ideal values of 109.5 (1) (Allen *et al.*, 1987). The corresponding bond angles of the parent Osthol molecule are 124.7 (2)° and 120.5 (2)° (Borowiak & Wolska (1989). The bond angle deviation at C7 is attributed to the attached propionamide group and the sp^2^ to sp^3^ hybridization change at C7 and C8 atoms. In the present structure the torsion angles C13—C7—C8—C9 and C14—C7—C8—C9 are -64.2 (2)° and 58.4 (2)°, respectively. The corresponding torsion angles in osthol are -1.61 (2)° and -177.8 (2)° (Borowiak & Wolska, 1989). The change in torsion angles is mainly due to the propionamide substitution at C7. C17 and C18 are disordered over two positions with refined occupancies of 0.69 (1) and 0.31 (1). The packing of the molecules is stabilized by the N1—H1N···O4 hydrogen bond, which forms an infinite chain in head to tail mode, running parallel to the c-direction (Fig. 2).

### Experimental

Propylnitrile was added to a solution of Osthol (100 mg) dissolved in benzene, and the reaction mixture was cooled at 0°c, followed by slow addition of sulfuric acid. The reaction mixture was stirred at room temperature for 2 to 3 hrs and was quenched after a complete disappearance of Osthol with a saturated solution of sodium bicarbonate. The final compound was extracted with ethyl acetate and dried over sodium sulfate. The compound was purified using column chromatography with 10% ethyl acetate in hexane as a mobile phase. Rod-shaped crystals were obtained using a slow-evaporation technique from a mixture of ethyl acetate and hexane.

### Refinement

The H atom bonded to N was freely refined. H atoms bonded to C were positioned geometrically (C—H = 0.93 - 0.98 Å) and allowed to ride on their parent atoms, with *U*_iso_(H) = 1.5 U_eq_(C) for methyl H and 1.2 U_eq_(C) for other H atoms. The C17 and C18 atoms are disordered over two positions (C17A/C17B and C18A/C18B) with refined occupancies of 0.69 (1) and 0.31 (1).

### Crystal data

**Table:**

C_18_H_23_NO_4_	*F*(000) = 680
*M**_r_* = 317.37	*D*_x_ = 1.229 Mg m^−^^3^
Monoclinic, *P*2_1_/*c*	Mo *K*α radiation, λ = 0.71073 Å
Hall symbol: -P 2ybc	Cell parameters from 6500 reflections
*a* = 11.3555 (11) Å	θ = 1.8–28.0°
*b* = 15.5452 (15) Å	µ = 0.09 mm^−^^1^
*c* = 9.7642 (10) Å	*T* = 273 K
β = 95.617 (2)°	Rod, yellow
*V* = 1715.3 (3) Å^3^	0.22 × 0.20 × 0.20 mm
*Z* = 4	

### Data collection

**Table:**

Bruker SMART APEX CCD area-detector diffractometer	3019 independent reflections
Radiation source: fine-focus sealed tube	2537 reflections with *I* > 2σ(*I*)
graphite	*R*_int_ = 0.019
ω scans	θ_max_ = 25.0°, θ_min_ = 1.8°
Absorption correction: multi-scan (*SADABS*; Sheldrick, 1996)	*h* = −13→13
*T*_min_ = 0.981, *T*_max_ = 0.983	*k* = −18→18
16205 measured reflections	*l* = −11→11

### Refinement

**Table:**

Refinement on *F*^2^	Primary atom site location: structure-invariant direct methods
Least-squares matrix: full	Secondary atom site location: difference Fourier map
*R*[*F*^2^ > 2σ(*F*^2^)] = 0.045	Hydrogen site location: inferred from neighbouring sites
*wR*(*F*^2^) = 0.136	H atoms treated by a mixture of independent and constrained refinement
*S* = 1.01	*w* = 1/[σ^2^(*F*_o_^2^) + (0.0736*P*)^2^ + 0.2891*P*] where *P* = (*F*_o_^2^ + 2*F*_c_^2^)/3
3019 reflections	(Δ/σ)_max_ = 0.007
234 parameters	Δρ_max_ = 0.16 e Å^−^^3^
0 restraints	Δρ_min_ = −0.13 e Å^−^^3^

### Special details

**Table:**

Geometry. All e.s.d.'s (except the e.s.d. in the dihedral angle between two l.s. planes) are estimated using the full covariance matrix. The cell e.s.d.'s are taken into account individually in the estimation of e.s.d.'s in distances, angles and torsion angles; correlations between e.s.d.'s in cell parameters are only used when they are defined by crystal symmetry. An approximate (isotropic) treatment of cell e.s.d.'s is used for estimating e.s.d.'s involving l.s. planes.
Refinement. Refinement of *F*^2^ against ALL reflections. The weighted *R*-factor *wR* and goodness of fit *S* are based on *F*^2^, conventional *R*-factors *R* are based on *F*, with *F* set to zero for negative *F*^2^. The threshold expression of *F*^2^ > σ(*F*^2^) is used only for calculating *R*-factors(gt) *etc*. and is not relevant to the choice of reflections for refinement. *R*-factors based on *F*^2^ are statistically about twice as large as those based on *F*, and *R*- factors based on ALL data will be even larger.

### Fractional atomic coordinates and isotropic or equivalent isotropic displacement parameters (Å^2^)

**Table:**

	*x*	*y*	*z*	*U*_iso_*/*U*_eq_	Occ. (<1)
C1	0.50266 (14)	0.38430 (10)	0.50309 (19)	0.0668 (4)	
C2	0.40239 (14)	0.37094 (10)	0.41396 (19)	0.0670 (4)	
C3	0.49068 (17)	0.37660 (11)	0.6435 (2)	0.0781 (5)	
C4	0.29202 (14)	0.35187 (11)	0.4587 (2)	0.0768 (5)	
C5	0.3822 (2)	0.35721 (13)	0.6915 (2)	0.0911 (6)	
H5	0.3758	0.3524	0.7854	0.109*	
C6	0.28580 (19)	0.34547 (12)	0.5993 (3)	0.0904 (6)	
H6	0.2134	0.3327	0.6317	0.108*	
C7	0.80188 (12)	0.36325 (10)	0.33969 (14)	0.0560 (4)	
C8	0.69360 (12)	0.33546 (10)	0.41152 (16)	0.0604 (4)	
H8A	0.6452	0.2972	0.3510	0.072*	
H8B	0.7203	0.3035	0.4941	0.072*	
C9	0.61786 (13)	0.41122 (10)	0.4504 (2)	0.0728 (5)	
H9A	0.6629	0.4451	0.5207	0.087*	
H9B	0.6006	0.4477	0.3703	0.087*	
C10	0.5809 (3)	0.4006 (2)	0.8721 (3)	0.1361 (10)	
H10A	0.5271	0.4466	0.8866	0.204*	
H10B	0.6573	0.4135	0.9186	0.204*	
H10C	0.5518	0.3480	0.9078	0.204*	
C11	0.19411 (17)	0.34260 (14)	0.3580 (3)	0.0988 (7)	
H11	0.1200	0.3300	0.3858	0.119*	
C12	0.3184 (2)	0.37156 (14)	0.1768 (3)	0.0996 (6)	
C13	0.76340 (19)	0.40583 (15)	0.20249 (19)	0.0922 (6)	
H13A	0.8313	0.4153	0.1532	0.138*	
H13B	0.7262	0.4599	0.2182	0.138*	
H13C	0.7083	0.3692	0.1494	0.138*	
C14	0.88212 (14)	0.42377 (11)	0.42793 (19)	0.0723 (4)	
H14A	0.9062	0.3968	0.5146	0.108*	
H14B	0.8402	0.4760	0.4433	0.108*	
H14C	0.9507	0.4368	0.3816	0.108*	
C15	0.2058 (2)	0.35152 (16)	0.2258 (3)	0.1089 (8)	
H15	0.1397	0.3447	0.1625	0.131*	
C16	0.92284 (12)	0.22894 (10)	0.39309 (13)	0.0563 (4)	
O1	0.41391 (11)	0.37975 (8)	0.27610 (13)	0.0811 (4)	
O3	0.3391 (2)	0.38160 (14)	0.0601 (2)	0.1400 (7)	
O2	0.59071 (14)	0.39123 (11)	0.72856 (16)	0.1065 (5)	
O4	0.92528 (10)	0.23463 (8)	0.51856 (9)	0.0719 (3)	
N1	0.86888 (10)	0.28549 (8)	0.30587 (12)	0.0572 (3)	
H1N	0.8755 (14)	0.2776 (11)	0.2195 (19)	0.069 (5)*	
C18A	0.9166 (6)	0.0731 (2)	0.3336 (6)	0.106 (2)	0.689 (12)
H18A	0.9576	0.0272	0.2928	0.159*	0.689 (12)
H18B	0.8408	0.0816	0.2826	0.159*	0.689 (12)
H18C	0.9056	0.0586	0.4271	0.159*	0.689 (12)
C17A	0.9890 (10)	0.1556 (8)	0.3311 (7)	0.0664 (16)	0.689 (12)
H17A	1.0653	0.1475	0.3833	0.080*	0.689 (12)
H17B	1.0023	0.1696	0.2370	0.080*	0.689 (12)
C17B	0.962 (2)	0.1530 (19)	0.316 (2)	0.094 (7)	0.311 (12)
H17C	0.8942	0.1293	0.2609	0.112*	0.311 (12)
H17D	1.0179	0.1725	0.2533	0.112*	0.311 (12)
C18B	1.013 (3)	0.0901 (11)	0.3947 (12)	0.217 (12)	0.311 (12)
H18D	1.0485	0.0490	0.3376	0.326*	0.311 (12)
H18E	0.9547	0.0620	0.4433	0.326*	0.311 (12)
H18F	1.0734	0.1142	0.4596	0.326*	0.311 (12)

### Atomic displacement parameters (Å^2^)

**Table:**

	*U*^11^	*U*^22^	*U*^33^	*U*^12^	*U*^13^	*U*^23^
C1	0.0561 (9)	0.0560 (9)	0.0928 (11)	0.0061 (7)	0.0297 (8)	0.0001 (7)
C2	0.0595 (9)	0.0552 (9)	0.0911 (11)	0.0046 (7)	0.0312 (8)	−0.0009 (8)
C3	0.0759 (11)	0.0680 (10)	0.0935 (13)	0.0148 (8)	0.0243 (10)	0.0045 (9)
C4	0.0552 (9)	0.0590 (9)	0.1213 (15)	0.0010 (7)	0.0339 (9)	−0.0018 (9)
C5	0.0998 (15)	0.0776 (12)	0.1041 (15)	0.0141 (11)	0.0519 (13)	0.0157 (10)
C6	0.0769 (12)	0.0717 (11)	0.1323 (18)	0.0023 (9)	0.0591 (13)	0.0095 (11)
C7	0.0524 (7)	0.0659 (9)	0.0521 (8)	0.0066 (6)	0.0162 (6)	0.0041 (6)
C8	0.0505 (8)	0.0604 (9)	0.0728 (9)	−0.0003 (6)	0.0185 (6)	−0.0053 (7)
C9	0.0539 (8)	0.0630 (9)	0.1058 (13)	−0.0010 (7)	0.0306 (8)	−0.0077 (9)
C10	0.178 (3)	0.138 (2)	0.0892 (16)	0.026 (2)	0.0005 (17)	0.0000 (15)
C11	0.0605 (11)	0.0806 (13)	0.158 (2)	−0.0012 (9)	0.0242 (13)	−0.0194 (14)
C12	0.1143 (18)	0.0812 (14)	0.1025 (17)	0.0039 (12)	0.0068 (14)	−0.0089 (11)
C13	0.1019 (14)	0.1103 (15)	0.0671 (10)	0.0359 (12)	0.0217 (9)	0.0226 (10)
C14	0.0611 (9)	0.0663 (10)	0.0919 (12)	−0.0056 (7)	0.0205 (8)	−0.0035 (8)
C15	0.0800 (14)	0.0943 (16)	0.149 (2)	0.0048 (11)	−0.0057 (15)	−0.0210 (15)
C16	0.0574 (8)	0.0676 (9)	0.0459 (7)	0.0040 (7)	0.0158 (6)	0.0000 (6)
O1	0.0775 (8)	0.0793 (8)	0.0902 (9)	−0.0017 (6)	0.0264 (7)	−0.0045 (6)
O3	0.179 (2)	0.1423 (16)	0.0978 (12)	−0.0107 (14)	0.0076 (12)	−0.0032 (11)
O2	0.0973 (11)	0.1188 (12)	0.1031 (11)	0.0189 (9)	0.0086 (8)	−0.0041 (9)
O4	0.0924 (8)	0.0822 (8)	0.0434 (6)	0.0163 (6)	0.0180 (5)	0.0032 (5)
N1	0.0585 (7)	0.0747 (8)	0.0404 (6)	0.0101 (6)	0.0143 (5)	−0.0015 (5)
C18A	0.156 (4)	0.071 (2)	0.100 (3)	0.010 (2)	0.064 (3)	−0.0056 (19)
C17A	0.065 (3)	0.089 (3)	0.0471 (18)	0.026 (2)	0.017 (2)	0.0018 (19)
C17B	0.106 (16)	0.082 (8)	0.093 (10)	0.020 (9)	0.012 (7)	−0.009 (6)
C18B	0.37 (3)	0.181 (13)	0.092 (7)	0.187 (18)	−0.003 (11)	−0.013 (7)

### Geometric parameters (Å, °)

**Table:**

C1—C2	1.379 (3)	C12—O3	1.196 (3)
C1—C3	1.396 (3)	C12—O1	1.388 (3)
C1—C9	1.511 (2)	C12—C15	1.443 (4)
C2—O1	1.372 (2)	C13—H13A	0.9600
C2—C4	1.399 (2)	C13—H13B	0.9600
C3—O2	1.359 (2)	C13—H13C	0.9600
C3—C5	1.393 (3)	C14—H14A	0.9600
C4—C6	1.385 (3)	C14—H14B	0.9600
C4—C11	1.418 (3)	C14—H14C	0.9600
C5—C6	1.360 (3)	C15—H15	0.9300
C5—H5	0.9300	C16—O4	1.2260 (16)
C6—H6	0.9300	C16—N1	1.3310 (19)
C7—N1	1.4827 (19)	C16—C17B	1.49 (3)
C7—C14	1.518 (2)	C16—C17A	1.523 (10)
C7—C13	1.520 (2)	N1—H1N	0.863 (18)
C7—C8	1.5359 (19)	C18A—C17A	1.525 (13)
C8—C9	1.528 (2)	C18A—H18A	0.9600
C8—H8A	0.9700	C18A—H18B	0.9600
C8—H8B	0.9700	C18A—H18C	0.9600
C9—H9A	0.9700	C17A—H17A	0.9700
C9—H9B	0.9700	C17A—H17B	0.9700
C10—O2	1.424 (3)	C17B—C18B	1.34 (3)
C10—H10A	0.9600	C17B—H17C	0.9700
C10—H10B	0.9600	C17B—H17D	0.9700
C10—H10C	0.9600	C18B—H18D	0.9600
C11—C15	1.318 (3)	C18B—H18E	0.9600
C11—H11	0.9300	C18B—H18F	0.9600
			
C2—C1—C3	116.94 (15)	C7—C13—H13B	109.5
C2—C1—C9	121.03 (16)	H13A—C13—H13B	109.5
C3—C1—C9	121.94 (17)	C7—C13—H13C	109.5
O1—C2—C1	116.87 (14)	H13A—C13—H13C	109.5
O1—C2—C4	120.14 (17)	H13B—C13—H13C	109.5
C1—C2—C4	122.96 (17)	C7—C14—H14A	109.5
O2—C3—C5	123.00 (19)	C7—C14—H14B	109.5
O2—C3—C1	115.46 (16)	H14A—C14—H14B	109.5
C5—C3—C1	121.5 (2)	C7—C14—H14C	109.5
C6—C4—C2	117.33 (18)	H14A—C14—H14C	109.5
C6—C4—C11	124.59 (18)	H14B—C14—H14C	109.5
C2—C4—C11	118.1 (2)	C11—C15—C12	121.7 (2)
C6—C5—C3	119.19 (19)	C11—C15—H15	119.2
C6—C5—H5	120.4	C12—C15—H15	119.2
C3—C5—H5	120.4	O4—C16—N1	123.76 (13)
C5—C6—C4	122.04 (16)	O4—C16—C17B	125.6 (9)
C5—C6—H6	119.0	N1—C16—C17B	110.0 (10)
C4—C6—H6	119.0	O4—C16—C17A	119.2 (3)
N1—C7—C14	109.82 (12)	N1—C16—C17A	117.0 (3)
N1—C7—C13	105.56 (12)	C17B—C16—C17A	12.4 (15)
C14—C7—C13	109.59 (15)	C2—O1—C12	122.22 (16)
N1—C7—C8	108.88 (12)	C3—O2—C10	118.5 (2)
C14—C7—C8	112.21 (12)	C16—N1—C7	127.60 (11)
C13—C7—C8	110.56 (13)	C16—N1—H1N	116.9 (11)
C9—C8—C7	113.07 (12)	C7—N1—H1N	115.4 (11)
C9—C8—H8A	109.0	C17A—C18A—H18A	109.5
C7—C8—H8A	109.0	C17A—C18A—H18B	109.5
C9—C8—H8B	109.0	H18A—C18A—H18B	109.5
C7—C8—H8B	109.0	C17A—C18A—H18C	109.5
H8A—C8—H8B	107.8	H18A—C18A—H18C	109.5
C1—C9—C8	113.47 (13)	H18B—C18A—H18C	109.5
C1—C9—H9A	108.9	C16—C17A—C18A	109.6 (6)
C8—C9—H9A	108.9	C16—C17A—H17A	109.8
C1—C9—H9B	108.9	C18A—C17A—H17A	109.8
C8—C9—H9B	108.9	C16—C17A—H17B	109.8
H9A—C9—H9B	107.7	C18A—C17A—H17B	109.8
O2—C10—H10A	109.5	H17A—C17A—H17B	108.2
O2—C10—H10B	109.5	C18B—C17B—C16	115.0 (16)
H10A—C10—H10B	109.5	C18B—C17B—H17C	108.5
O2—C10—H10C	109.5	C16—C17B—H17C	108.5
H10A—C10—H10C	109.5	C18B—C17B—H17D	108.5
H10B—C10—H10C	109.5	C16—C17B—H17D	108.5
C15—C11—C4	121.4 (2)	H17C—C17B—H17D	107.5
C15—C11—H11	119.3	C17B—C18B—H18D	109.5
C4—C11—H11	119.3	C17B—C18B—H18E	109.5
O3—C12—O1	116.1 (2)	H18D—C18B—H18E	109.5
O3—C12—C15	127.4 (3)	C17B—C18B—H18F	109.5
O1—C12—C15	116.5 (2)	H18D—C18B—H18F	109.5
C7—C13—H13A	109.5	H18E—C18B—H18F	109.5
			
C3—C1—C2—O1	179.09 (13)	C6—C4—C11—C15	178.4 (2)
C9—C1—C2—O1	2.6 (2)	C2—C4—C11—C15	0.0 (3)
C3—C1—C2—C4	1.0 (2)	C4—C11—C15—C12	−0.3 (4)
C9—C1—C2—C4	−175.40 (14)	O3—C12—C15—C11	−179.5 (2)
C2—C1—C3—O2	−179.19 (14)	O1—C12—C15—C11	0.9 (3)
C9—C1—C3—O2	−2.8 (2)	C1—C2—O1—C12	−177.17 (15)
C2—C1—C3—C5	−0.7 (2)	C4—C2—O1—C12	0.9 (2)
C9—C1—C3—C5	175.75 (16)	O3—C12—O1—C2	179.15 (18)
O1—C2—C4—C6	−178.77 (15)	C15—C12—O1—C2	−1.2 (3)
C1—C2—C4—C6	−0.8 (2)	C5—C3—O2—C10	−10.2 (3)
O1—C2—C4—C11	−0.3 (2)	C1—C3—O2—C10	168.33 (18)
C1—C2—C4—C11	177.66 (16)	O4—C16—N1—C7	−0.9 (2)
O2—C3—C5—C6	178.46 (18)	C17B—C16—N1—C7	170.6 (12)
C1—C3—C5—C6	0.0 (3)	C17A—C16—N1—C7	−178.2 (5)
C3—C5—C6—C4	0.2 (3)	C14—C7—N1—C16	59.89 (19)
C2—C4—C6—C5	0.1 (3)	C13—C7—N1—C16	177.95 (16)
C11—C4—C6—C5	−178.22 (19)	C8—C7—N1—C16	−63.33 (18)
N1—C7—C8—C9	−179.76 (13)	O4—C16—C17A—C18A	78.8 (7)
C14—C7—C8—C9	58.45 (18)	N1—C16—C17A—C18A	−103.7 (5)
C13—C7—C8—C9	−64.23 (19)	C17B—C16—C17A—C18A	−46 (6)
C2—C1—C9—C8	−87.9 (2)	O4—C16—C17B—C18B	−6(3)
C3—C1—C9—C8	95.79 (19)	N1—C16—C17B—C18B	−177 (2)
C7—C8—C9—C1	172.05 (14)	C17A—C16—C17B—C18B	56 (5)

### Hydrogen-bond geometry (Å, °)

**Table:**

*D*—H···*A*	*D*—H	H···*A*	*D*···*A*	*D*—H···*A*
N1—H1N···O4^i^	0.87 (2)	2.10 (2)	2.9546 (15)	169.1 (15)

Symmetry codes: (i) *x*, −*y*+1/2, *z*−1/2.
